# Twenty-one-year follow-up revealed guideline-concordant and non-concordant trends in intensive care of bronchiolitis

**DOI:** 10.1007/s00431-023-04940-2

**Published:** 2023-03-29

**Authors:** Sofia Selin, Minna Mecklin, Matti Korppi, Paula Heikkilä

**Affiliations:** 1 Faculty of Medicine and Health Technology, Tampere University, Tampere, Finland; 2 Tampere Centre for Child, Adolescent and Maternal Health Research, Faculty of Medicine and Health Technology, Tampere University and Tampere University Hospital, Tampere, Finland

**Keywords:** Bronchiolitis, Intensive care, Bronchodilators, Respiratory support, Guideline

## Abstract

To evaluate the management of bronchiolitis in the paediatric intensive care unit (PICU) before and after publication of the national bronchiolitis guidelines in June 2015. All infants treated between 2016–2020 for bronchiolitis in the PICU of Tampere University Hospital at < 12 months of age were included. The data were retrospectively collected from electronic patient records. The current results reflecting the post-guideline era were compared with previously published results for the pre-guideline 2000–2015 period. These two studies used identical protocols. Forty-six infants treated in the PICU were included. During the post-guideline era, inhaled adrenaline was given to 26 (57%), salbutamol to 7 (15%), and hypertonic saline inhalations to 35 (75%) patients. Forty-three patients (94%) received high-flow oxygen therapy (HFOT). Seventeen patients (37%) were treated with nasal continuous positive airway pressure (CPAP) and 4 (9%) with mechanical ventilation.

*Conclusion*: When post-guideline years were compared with pre-guideline years, the use of bronchodilators decreased in agreement, but the use of inhaled saline increased in disagreement with the guidelines. The use of respiratory support increased, evidently because of an introduction of the non-invasive HFOT treatment modality.

**Table:** 

**What is Known:** *• Oxygen supplementation and respiratory support, when needed, are the cornerstones of bronchiolitis treatment.* *• Medicines are frequently given to infants with bronchiolitis, especially if intensive care is needed, although evidence of their effectiveness is lacking.*	
**What is New:** *• Nearly all (94%) infants who needed intensive care were treated with HFOT and 37% with nasal CPAP, and finally, only 9% were intubated, which reflects the effectiveness of non-invasive techniques.* *• When pre- and post-guideline eras were compared, use of racemic adrenaline decreased from 84 to 57%, but use of hypertonic saline increased up to 75%, which disagrees with the current guidelines.*

**What is Known:**

*• Oxygen supplementation and respiratory support, when needed, are the cornerstones of bronchiolitis treatment.*

*• Medicines are frequently given to infants with bronchiolitis, especially if intensive care is needed, although evidence of their effectiveness is lacking.*

**What is New:**

*• Nearly all (94%) infants who needed intensive care were treated with HFOT and 37% with nasal CPAP, and finally, only 9% were intubated, which reflects the effectiveness of non-invasive techniques.*

*• When pre- and post-guideline eras were compared, use of racemic adrenaline decreased from 84 to 57%, but use of hypertonic saline increased up to 75%, which disagrees with the current guidelines.*

## Introduction

Bronchiolitis is the first wheezing episode at < 12 months of age induced by viral lower respiratory tract infection [1]. In total, 2%–3% of infants are treated in hospital for bronchiolitis [2] and in a Finnish population-based study, 6% of those who visited the paediatric emergency department (ED), were treated in the paediatric intensive care unit (PICU) [3]. The most important risk factors for severe disease and PICU admission are young age of < 2 months, birth weight of < 2000 g, congenital heart disease, chronic lung disease, immunocompromised state and being hypotonic [4–6].

There is no specific curative treatment for bronchiolitis, but oxygen supplementation and respiratory and feeding supports are often needed [1]. The Finnish Current Care Guideline for bronchiolitis recommends high-flow nasal oxygenation therapy (HFOT) with warmed and humified oxygen-air mixture when standard low-flow oxygen supplementation is insufficient [7]. There is preliminary evidence from retrospective studies that HFOT may reduce the need for mechanical ventilation [8–10]. Instead, no medicines are recommended for bronchiolitis. Antibiotics should not be used since bronchiolitis is a viral infection with a low prevalence of secondary bacterial infections [11]. Inhaled bronchodilators, such as beta-agonists, racemic adrenaline, and systemic or inhaled corticosteroids, are not recommended due to a lack of research-based evidence on their effectiveness in bronchiolitis [7, 11, 12]. The use of hypertonic saline is controversial, since after initial promising results, recent studies have failed to show any benefits in bronchiolitis [13, 14].

The Finnish Current Care Guidelines for lower respiratory tract infections in children, including recommendations for infant bronchiolitis, were published in June 2015 [7], and they are in line with the international evidence-based guidelines [1, 15, 16]. The upper age limit of bronchiolitis is 12 months in European and 24 months in American guidelines; otherwise, the recommendations are rather similar. We have previously published the observations on bronchiolitis treatment in the PICU of our university hospital for 2000–2015 [17], which represents the era before the Finnish Current Care Guidelines. The aim of this retrospective descriptive study was to evaluate bronchiolitis treatment in the PICU of the same hospital in 2016–2020, which represents the post-guideline era. The identical designs of the present and previous studies make it possible to compare the pre-guideline and post-guideline treatments. The long surveillance time allows for evaluating of the trends for 20 years.

## Methods

### Study design and seating

This descriptive, retrospective study reviewed the registered data of all patients who were treated for bronchiolitis at age < 12 months between 1 January 2016 and 31 December 2020 in the PICU of Tampere University Hospital, Tampere, Finland. The hospital provided secondary care for a population of approximately 4,400 infants < 12 months of age, and tertiary care for a population of nearly 7,100 infants < 12 months of age, in 2020, the last year of our surveillance period [18].

### Sample, inclusion, and exclusion criteria

We included in the study all infants aged < 12 months and diagnosed with bronchiolitis and treated in the PICU. Bronchiolitis was defined as the first wheezing-associated, presumably viral lower respiratory infection. Those who were admitted to the PICU with bronchiolitis as the primary reason were included in the study. We excluded those infants who were admitted to the PICU for another primary reason, such as pneumonia.

### Data collection

We identified from the electronic patient files of Tampere University Hospital all infants who were treated at < 12 months of age with ICD-10 codes J10*-18*, J20*-22*, J45* and J46*. Thereafter, medical records were reviewed by one of the authors (PH). The data collection was identical to that performed for 2000–2015 and published previously [17].

Seventy-six infants were identified based on the ICD-10 codes, and 30 of them were excluded according to our exclusion criteria, since bronchiolitis was not the primary reason for PICU admission. Eighteen of them had pneumonia, two had another infection, and ten were treated in the PICU for reasons such as postoperative follow-up or neurological problems. Five cases recorded as bronchiolitis were excluded because they had been treated in hospital for bronchiolitis previously, and thus, did not fulfil the diagnostic criterion of the first episode.

### Variables

Information on the medical care of the patient was obtained from the electronic patient files, and one of the authors (SS) checked the recordings and collected the available data in a structured form. Data was collected for the stays in the emergency department (ED), the ward and the PICU. Medical history included gestational age in weeks, underlying diseases, observed allergies and consumed medicines. For the period in hospital, data on clinical findings, chest radiograph findings and given medical treatments were collected. Clinical findings were collected separately for the ED, the ward and the PICU and comprised data, for example, on lung auscultation, the lowest oxygen saturation, fever (both categorially defined as ≥ 38 degree and the highest value), and signs of dehydration. Either a respiratory syncytial virus (RSV) antigen test or a panel of polymerase chain reactions for respiratory viruses (including RSV and rhinovirus) were studied during hospitalisation according to clinical practice. Oxygen support was categorially recorded separately for low-flow and high-flow treatment modalities. In the case of HFOT, length of use in days was also recorded. In addition, the use (yes or no) was collected for the following treatments: enteral or parenteral fluid supports, physiologic or hypertonic saline inhalations, and administrations of inhaled adrenaline, salbutamol, anticholinergics or systemic or inhaled corticosteroids during hospitalisation, and magnesium sulphate infusions in the PICU. The use of non-invasive respiratory support such as nasal CPAP and invasive respiratory support requiring intubation were collected both categorially and in days. The length of stay (LOS) in days was registered for both the ward and PICU treatment periods. The LOS in hospital and in the PICU were compared with previously published data between 2000–2015. To evaluate the changes in the LOS in hospital and in the PICU, and the changes in different respiratory supports, we combined the identically collected data from 2000–2015 to current data from 2016–2020.

### Statistics

IBM SPSS Statistics, version 26, was used for data management. Continuous variables were presented as medians with either minimum and maximum or interquartile ranges (IQR). We used Mann–Whitney U test or Kruskal–Wallis test for non-normally distributed continuous variables. Categorised variables were presented as numbers and percentages and χ^2^ or Fisher’s exact tests were used. A two-sided p value < 0.05 was considered statistically significant.

### Ethics

The data were collected retrospectively from electronic patient files, and the patients or their guardians were not contacted. According to Finnish law, a statement is not needed from the Ethics Committee, and therefore, the study was conducted with the permission of the Head Doctor of Tampere University Hospital. The Declaration of Helsinki and good scientific practice were followed.

## Results

**Fig. 1:**
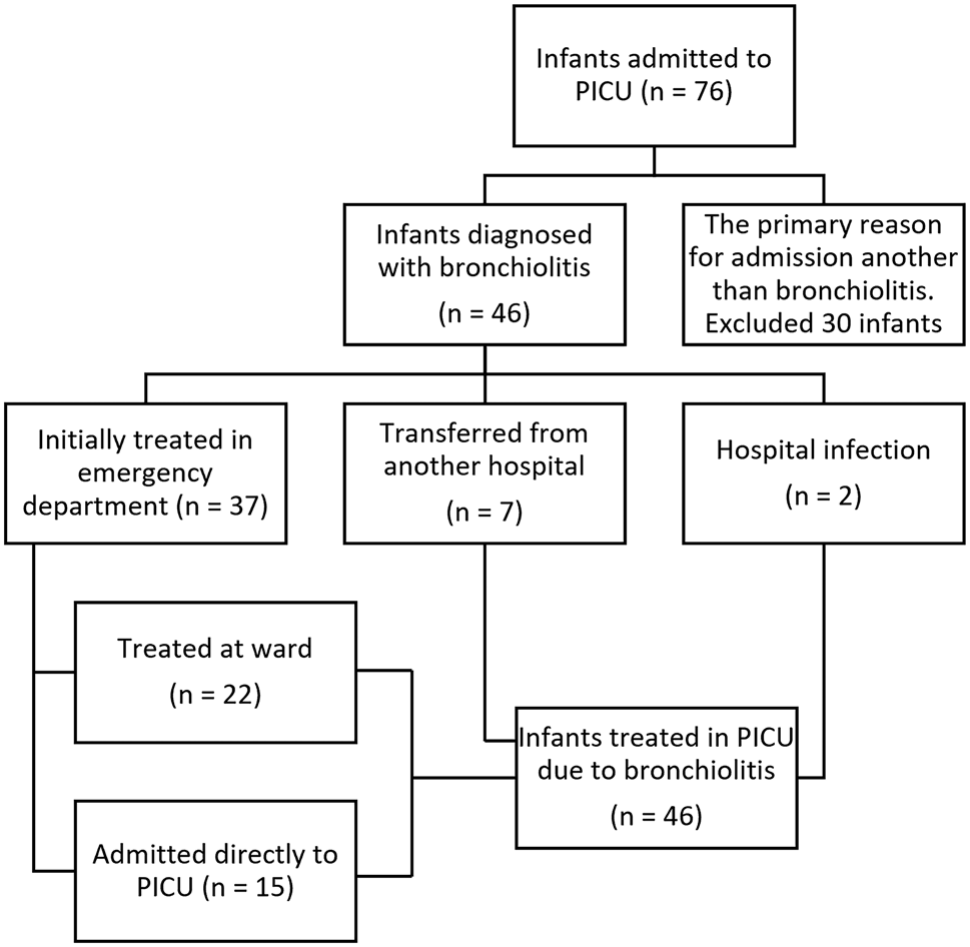
Flowchart of 46 infants diagnosed with bronchiolitis and treated in paediatric intensive care unit (PICU)

During this five-year surveillance period, 46 infants were treated for bronchiolitis at < 12 months of age in the PICU of Tampere University Hospital. Fifteen of them were admitted from the ED directly to the PICU, and 22 were first admitted to the paediatric ward (Fig. 1). Seven were admitted from another hospital of the tertiary care area to the university hospital and two infants had nosocomial infection and were admitted to the PICU from the ward.

The median age of patients on admission was 1.3 months (IQR 0.68–2.37), and 70% were boys. Eleven (24%) were born premature and a thirteen had needed hospital care after birth (Table 1). Only one patient was treated with palivizumab prophylaxis. Six infants had been diagnosed with congenital heart disease, such as ventricular septal defect, atrial septal defect or congenital stenosis of aortic valve, and four were diagnosed with lung disease, such as respiratory distress syndrome as new-born or bronchopulmonary dysplasia in infancy.

**Table 1:** Demographics of 46 infants with bronchiolitis treated in the paediatric intensive care unit between 2016 and 2020

	**n = 46 (%)**
Gender, boys	32 (70)
Age on admission in months, median (min–max)	1.3 (0.39–11.2)
Gestational age in weeks, median (min–max)	39 (23–41)
Need for hospital care after birth	13/44 (20)
Diagnosed with an underlying illness	9/41 (22)
Inhaled beta-agonist, before admission	2/40 (5)

Chest radiograph was taken from 40 (87%) infants: 27 (59%) during and 13 before PICU admission. Among them, 25 (63%) presented with an abnormal radiograph, and pulmonary atelectasis was the most common finding. None of the patients had pneumothorax. RSV was the most common etiological agent of bronchiolitis (89%), and six (13%) infants had rhinovirus.

During the PICU stay, fluid support was provided to 38 (83%) infants: via nasogastric tube in 14 (37%), intravenously in 7 (18%), and via both methods in 17 (45%) cases (Table 2). Inhaled bronchodilators were provided in the PICU to 29 (63%) infants, and 26 (57%) received inhaled adrenaline and 7 (15%) inhaled salbutamol. Two infants were treated with systemic steroids, and anticholinergics or magnesium sulphate were given to only one (2%). Hypertonic saline inhalations were given to 35 (76%) patients (Table 2).

**Table 2:** Management of infants with bronchiolitis in the paediatric intensive care unit (PICU), on the ward, and in the emergency department (ED)*

	**PICU n = 46** **n (%)**	**Ward n = 40** **n (%)**	ED n = 35** n (%)
Oxygen support	43 (94)	32 (80)	11 (31)
Fluid support p.o.	31 (67)	27 (68)	3 (9)
Fluid support i.v.	24 (52)	19 (48)	8 (23)
Inhaled			
Adrenaline	26 (57)	6 (15)	2 (6)
Beta-agonist	7 (15)	6 (15)	4 (11)
NaCl 0,9%	0	4 (10)	2 (6)
NaCl 3%	35 (76)	24 (60)	1 (3)
Ipratropiumbromid	1 (2)	0	0
Systemic steroids	2 (4)	1 (3)	1 (3)
Magnesium sulphate i.v.	1 (2)	0	0
HFOT	43 (94)	30 (75)	0
nCPAP	17 (37)	0	0
Mechanical ventilation	4 (9)	0	0

*HFOT* high-flow oxygen therapy, *i.v.* intravenous, *NaCl* sodium chloride, *nCPAP* nasal continuous positive airway pressure, *p.o.* per os

*From all 46 patients, there was also information collected and presented here of their possible treatment in the ED and ward, before or after being treated in the PICU

**The information of two patients is missing as they were transported straight to the PICU without actually being treated in the ED

Supplemental oxygen was given to 43 (94%) patients (Table 2). In total, 43 (94%) infants were treated with HFOT, the median length of treatment being 1.5 days (IQR 1–2.6). Nasal CPAP was used in 17 (37%) patients, with the median length of treatment being 2 days (range 0.5–2.5). Four (9%) patients were intubated and mechanically ventilated (Table 2).

When the four time periods of 2000–2005, 2006–2010, 2011–2015 [17] and 2016–2020 were compared, the use of inhaled salbutamol decreased, inhaled hypertonic saline increased, systemic steroids decreased, and HFOT increased (Table 3). The use of inhaled adrenaline and nasal CPAP first increased and then decreased. There were no constant changes in the trends of oral or intravenous fluid administrations, in obtaining chest radiographs, nor in median stays in the PICU or in hospital (Table 3).

**Table 3:** The change in in management of bronchiolitis in the paediatric intensive care unit (PICU) during 2000–2020

**Treatment**	**Years 2016–2020** **N = 46 (%)**	**Years 2011–2015** **N = 37 (%)**	**Years 2006–2010** **N = 27 (%)**	**Years 2000–2005** **N = 41 (%)**
Oxygen support	43 (94)^NS^	30 (81)	26 (96)	35 (85)
Fluid support (p.o.)	31 (67)^NS^	26 (70)	17 (63)	25 (61)
Fluid support (i.v.)	24 (52)^a^	29 (78)	21 (78)	24 (59)
Inhaled saline	35 (76)	21 (57)	6 (22)	2 (5)
0.9%	0^NS^	2/18 (11)	6 (22)	2 (5)
3%	35 (76)^b^	19/35 (54)	0/21	0/39
Inhaled bronchodilators	29 (63)	34 (92)	23 (85)	34 (83)
Adrenaline	26 (57)^NS^	31 (84)	21 (78)	24 (59)
Salbutamol	7 (15)^c^	12 (44)	14 (38)	28 (68)
Anticholinergic	1 (2)^NS^	0 (0)	2 (7)	6 (15)
Theophylline	0^NS^	3 (8)	1/26 (4)	4 (10)
Systemic steroids	2 (4)^d^	2 (5)	4 (15)	12 (29)
Radiography	27 (59)^NS^	24 (65)	20 (74)	26 (63)
HFOT	43 (94)^e^	26 (70)	1 (4)	0
nCPAP	17 (37)^NS^	21 (57)	12 (44)	5 (12)
Mechanical ventilation	4 (9)^NS^	7 (19)	7 (26)	7 (17)
Median LOS in PICU in days (IQR)^f^	3 (2–5.25)	4 (2–7.5)	2 (1–5)	3 (1–6.5)
Median LOS in hospital in days (IQR)^g^	6 (5–9)	8 (4–12.5)	5 (4–8)	7 (5–12)

*p = 0.055 for inhaled adrenaline*

*HFOT* high-flow oxygen therapy, *i.v.* intravenous, *nCPAP* nasal continuous positive airway pressure, *p.o.* per os

Statistical significance between years 2016–2020 and 2000–2015 (combined): ^a^p = 0.030, ^b^p < 0.001, ^c^p < 0.001, ^d^p < 0.001, ^e^p = 0.033, ^f^p = 0.344 and ^g^p = 0.102 between the groups, ^NS^Non-significant

The data from 2000–2015 (n = 105) was combined with current data from 2016–2020 (n = 46) and categorised based on respiratory support (Table 4). The median stays in hospital and in the PICU were calculated for each respiratory support-based group. The use of HFOT did not either increase or decrease stay in the PICU. The use of non-invasive and invasive respiratory support increased both median stays in hospital (5 and 11 days, respectively, p < 0.005) and in the PICU (4 and 6 days, respectively, p < 0.001) when compared to the PICU treated infants without respiratory support.

**Table 4:** Length of stay (LOS) in hospital and in the paediatric intensive care unit (PICU) of infants with bronchiolitis diagnosed between 2000 and 2020 presented as separately on the basis of respiratory support

**LOS**	**All infants** **n = 151**	**No respiratory support** **n = 54**	**HFOT** **n = 34**	**nCPAP** **n = 38**	**Mechanical ventilation** **n = 25**
In hospital in days, median (IQR)	6 (4–11)	5 (3.75–7)	6 (4–9)^a^	8 (5–11.25)^b^	11 (6.5–16)^c^
In the PICU in days, median (min–max)	3 (2–6)	2 (1–3)	2.5 (2–5)^NS^	4 (3–6)^d^	6 (4–10)^e^

*HFOT* high-flow oxygen therapy, *nCPAP* nasal continuous positive airway pressure, *NS* non-significant

^a^p 0.036, HFOT *vs*. no respiratory support

^b^p 0.003, nCPAP *vs*. no respiratory support

^c^p < 0.001, mechanical ventilation *vs*. no respiratory support

^d^p < 0.001, HFOT *vs*. no respiratory support

^e^p < 0.001, mechanical ventilation *vs*. no respiratory support

## Discussion

Two main results in the present study on the management of infants who needed intensive care for bronchiolitis were obvious. First, inhaled saline and bronchodilators were still used in 2016–2020 although the Finnish 2015 Current Care Guidelines did not recommend their use. Hypertonic saline inhalations were given to 76% of infants, and 57% received inhaled adrenaline and 15% inhaled salbutamol. The use of hypertonic saline increased, but in line with the guidelines, that of racemic adrenaline and salbutamol decreased gradually between 2000 and 2020. Second, the use of HFOT increased remarkably after 2015, and in 2016–2020, as many as 94% of infants were treated with HFOT. HFOT did not appear to influence the length of stay in hospital or in the PICU; however, the failure rates were quite low, since only 17% of infants needed nasal CPAP and 4% mechanical ventilation.

The use of inhaled hypertonic saline in the PICU increased over time. The figure was 22% in 2006–2010 and 57% in 2011–2015 [17], and increased to 76% in 2016–2020. The 2015 Current Care Guidelines stated that hypertonic saline inhalations do not apparently reduce the symptoms or the length of stay in hospital [7]. A cumulative meta-analysis showed that after the first positive results of hypertonic saline in bronchiolitis, the newer studies have been negative, and the cumulative benefits have changed to being marginal [19]. The National Institute for Health and Care Excellence (NICE) guidelines for the treatment of bronchiolitis were published in 2015 and updated in 2021 and recommended avoiding nebulised therapies, including hypertonic 3%–5% saline [15]. The effects of the NICE guidelines for bronchiolitis treatment were evaluated in 165 British infants treated in the PICU in 2011–2012 prior and in 187 infants treated in 2015–2016 after the guidelines were introduced [20]. In disagreement with the guidelines, the use of hypertonic saline increased from 8 to 29% and that of physiologic saline from 7 to 22%. The American Academy of Pediatrics (AAP) guidelines for bronchiolitis, published in 2014, recommended avoiding the use of hypertonic saline in the ED but allowed its use in hospitalised infants if necessary [1]. A Spanish study evaluated the effect of the AAP recommendations by comparing 340 infants treated in the PICU in 2010–2014 and 366 treated in 2015–2017 and found that the use of inhaled saline was rather similar in both periods, 59% and 62% respectively [21]. In the present study, the figures were higher than in the UK or Spain, but the increasing trends were similar in these three studies.

The 2015 Current Care Guidelines did not recommend using of bronchodilators, such as inhaled adrenaline or salbutamol [7]. On the other hand, the guidelines, which are focused on ED and ward settings, are not directly applicable for intensive care, which was the focus of the present study. A large multicentre, retrospective study that included 446,696 ED visits found that the use of bronchodilators did not reduce PICU admissions or the need for respiratory support [22]. We found that the use of both racemic adrenaline and salbutamol decreased in the PICU when compared to earlier study periods in 2000–2015 [17]. Adrenaline inhalations decreased from 84% in 2011–2015 to 57% in 2016–2020 and the use of salbutamol has continuously decreased from 68 to 15% during the 20 study years. These findings are in line with recent studies indicating a minor influence of the guidelines on the treatment of bronchiolitis [21]. In the PICU in Spain, the use of salbutamol was 35% in 2010–2014 and 33% in 2015–2017 in 706 infants, without any significant decrease [21].

The use of inhaled adrenaline varied more than that of salbutamol. In our previous study, the use in the PICU increased from 59% in 2000–2005 to 84% in 2011–2015 and then returned to 57% [17]. In the early 2000s, there were positive results in preliminary studies on adrenaline inhalations in bronchiolitis [23], but in the 2010s, the studies failed to show any benefits [24]. In Spain, the use of inhaled adrenaline for bronchiolitis in the PICU increased slightly from 46% in 2010–2014 in 340 patients to 55% in 2015–2017 in 366 patients [21]. The use of adrenaline inhalations has decreased, but inhaled adrenaline and salbutamol are still widely used in intensive care, as confirmed by the figure of 63% of infants in the present study.

The recommendation for bronchiolitis treatment in the 2015 Current Care Guidelines was to monitor and to support oxygenation, respiration and fluid intake when needed. HFOT has been proven to reduce treatment failures [25, 26], and nearly all (94%) patients treated in the PICU received HFOT in the present study. In Tampere University Hospital, HFOT was introduced for bronchiolitis treatment in 2010, and since then, its use has substantially increased in the PICU from 4% in 2010 to 70% in 2011–2015 [17] and further to 94% in 2016–2020. An active use of HFOT is in line with the 2015 Current Care Guidelines, and a major benefit is that HFOT can also be provided in the paediatric ED and ward. In 2011–2015, 57% of infants were treated with nasal CPAP [17], compared to 37% in 2016–2020, and at the same time, intubation rates decreased from 19 to 9%. Thus, the use of HFOT lessened the need for intensive care, which is laborious for infants and expensive for the community [27]. In line with the present study, a marked increase in the use of HFOT has been reported in England and Spain [20, 28], with a reduction in the need for non-invasive respiratory support [20].

In the present study, the stay both in the PICU and in hospital was found to be longer in infants treated with mechanical ventilation compared to those treated with nCPAP or HFOT. This clearly indicate more severe disease, but it may also indicate the unclear or varying criteria for PICU treatment. A previously published study introduced transfer criteria and standardization of transfer-readiness assessment for PICU-treated patients with bronchiolitis. In that study, reduced time-to-transfer decisions, and increased proportion of transfers with HFOT > 6 L/min were noticed. However, the stay in the PICU was not shortened [29]. This kind of guidelines and transfer criteria probably helps clinicians to evaluate more effectively the safe time to transfer patient from the PICU to the pediatric ward.

This study describes the management of bronchiolitis in the PICU and the impact of the bronchiolitis guidelines in intensive care, even though the guidelines were primarily targeted to outpatient and ward care. This study has some limitations. Although this was retrospective, register-based study with small sample, all patient recordings were also examined manually. The study design was identical with that of our previous 16-year study, allowing appropriate comparisons, and in addition, the combined data allowed us to evaluate treatment trends for 21 years. Since the study population (i.e., infants who need intensive care for bronchiolitis) was small, the applicability of this study is limited, and therefore, larger multicentre studies are needed. In addition, the question of whether infants treated for bronchiolitis in the PICU should have specific guidelines remains open.

In conclusion, this retrospective 5-year study on bronchiolitis treatment in the PICU revealed that bronchiolitis is still treated using methods that are not recommended by treatment guidelines.
